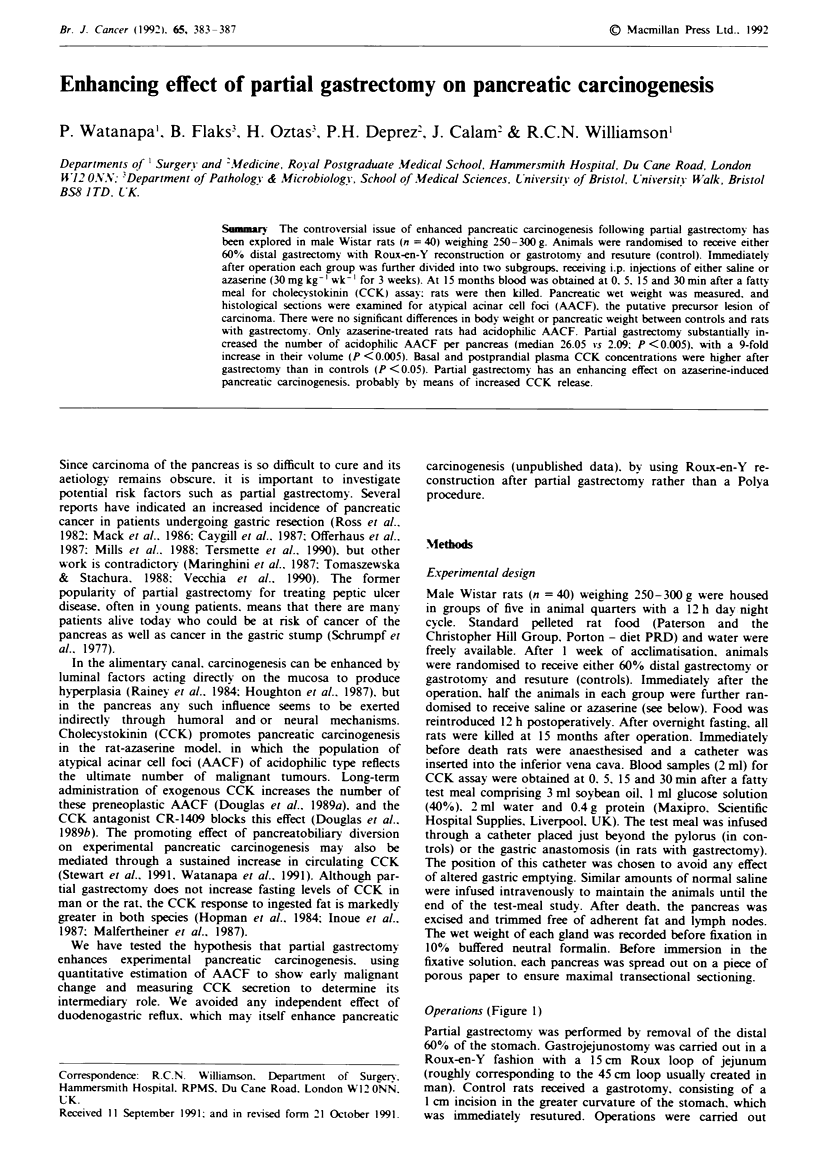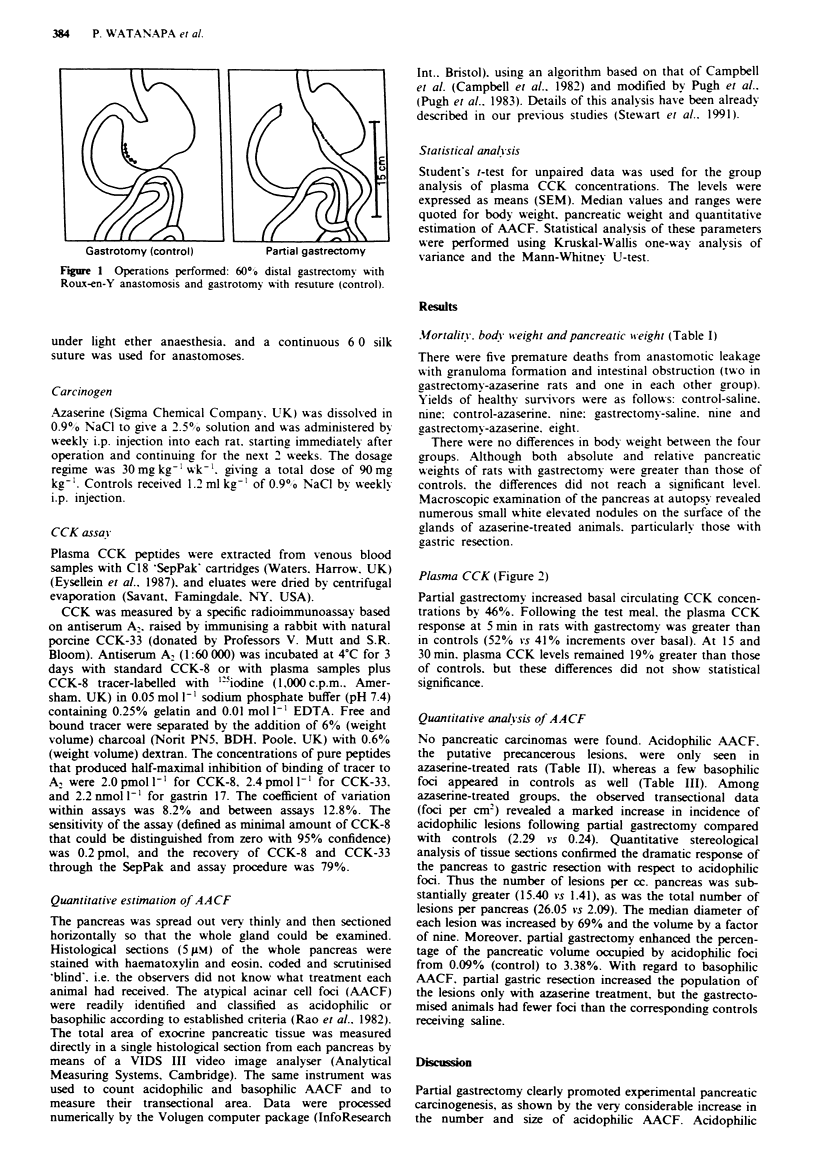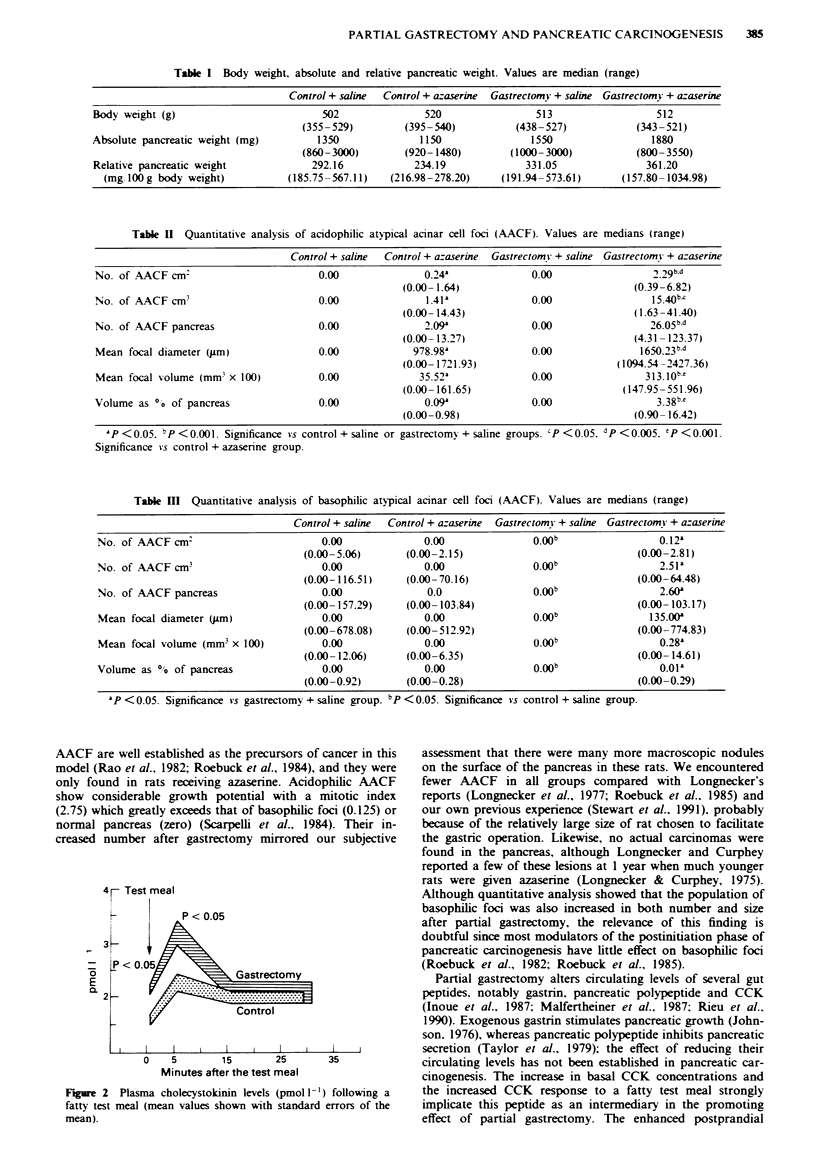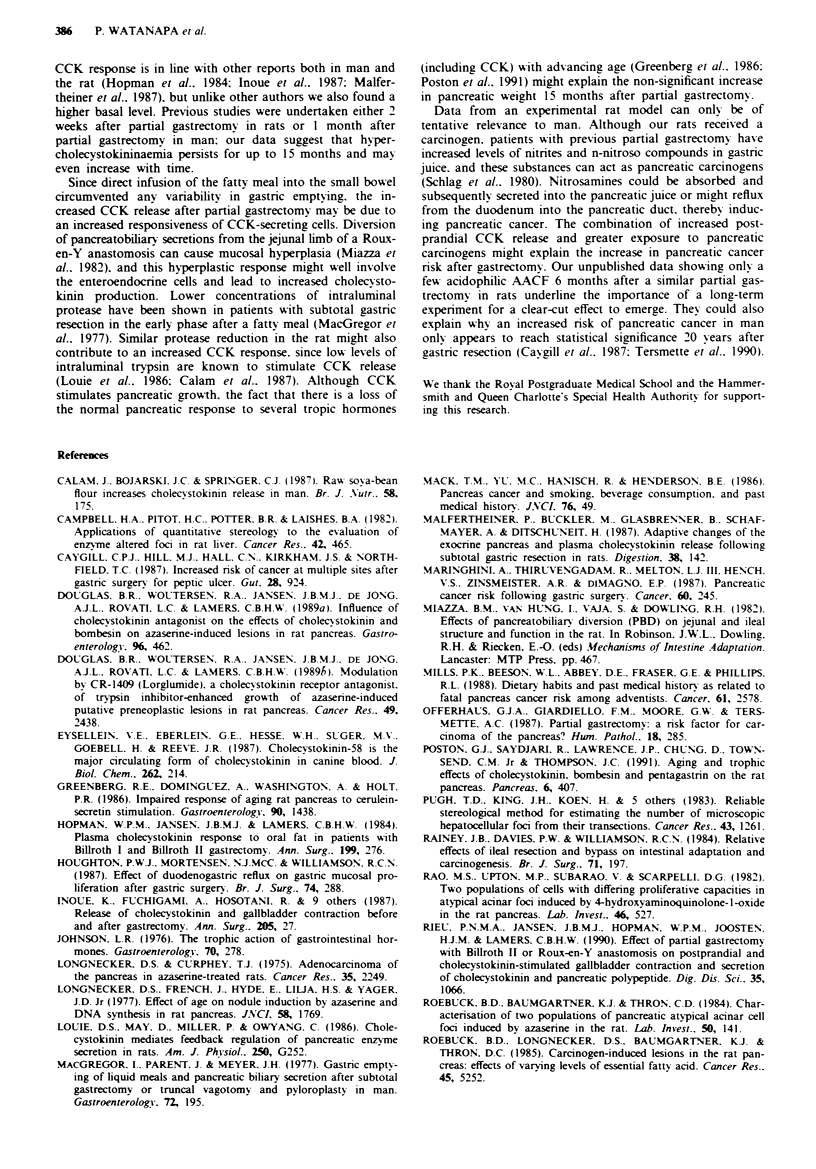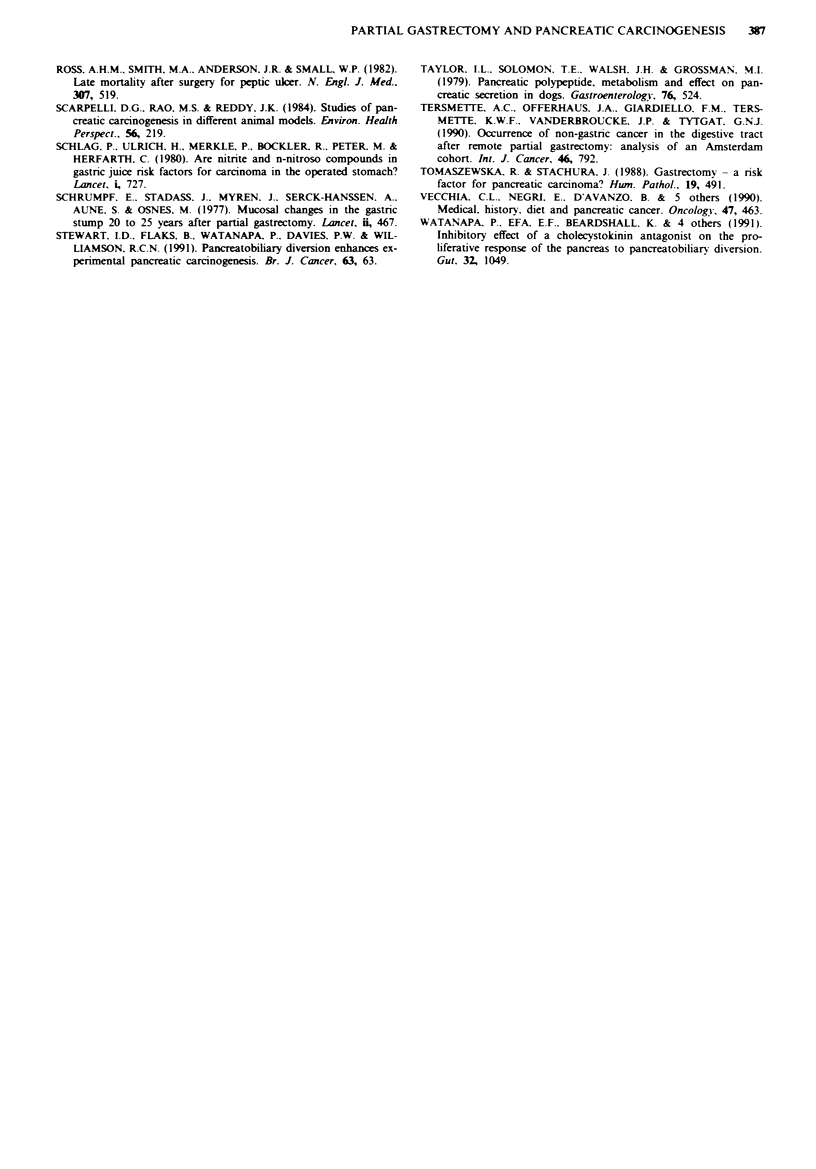# Enhancing effect of partial gastrectomy on pancreatic carcinogenesis.

**DOI:** 10.1038/bjc.1992.77

**Published:** 1992-03

**Authors:** P. Watanapa, B. Flaks, H. Oztas, P. H. Deprez, J. Calam, R. C. Williamson

**Affiliations:** Department of Surgery, Royal Postgraduate Medical School, Hammersmith Hospital, London, UK.

## Abstract

The controversial issue of enhanced pancreatic carcinogenesis following partial gastrectomy has been explored in male Wistar rats (n = 40) weighing 250-300 g. Animals were randomised to receive either 60% distal gastrectomy with Roux-en-Y reconstruction or gastrotomy and resuture (control). Immediately after operation each group was further divided into two subgroups, receiving i.p. injections of either saline or azaserine (30 mg kg-1 wk-1 for 3 weeks). At 15 months blood was obtained at 0, 5, 15 and 30 min after a fatty meal for cholecystokinin (CCK) assay; rats were then killed. Pancreatic wet weight was measured, and histological sections were examined for atypical acinar cell foci (AACF), the putative precursor lesion of carcinoma. There were no significant differences in body weight or pancreatic weight between controls and rats with gastrectomy. Only azaserine-treated rats had acidophilic AACF. Partial gastrectomy substantially increased the number of acidophilic AACF per pancreas (median 26.05 vs 2.09; P less than 0.005), with a 9-fold increase in their volume (P less than 0.005). Basal and postprandial plasma CCK concentrations were higher after gastrectomy than in controls (P less than 0.05). Partial gastrectomy has an enhancing effect on azaserine-induced pancreatic carcinogenesis, probably by means of increased CCK release.


Br. J. Cancer (1992), 65, 383–387 © Macmillan Press Ltd., 1992

### Enhancing effect of partial gastrectomy on pancreatic carcinogenesis

P. Watanapa[1], B. Flaks[3], H. Oztas[3], P.H. Deprez[2], J. Calam[2] & R.C.N. Williamson[1]

*Departments of [1] Surgery and [2]Medicine, Royal Postgraduate Medical School, Hammersmith Hospital, Du Cane Road, London W12 0NN; [3]Department of Pathology & Microbiology, School of Medical Sciences, University of Bristol, University Walk, Bristol BS8 1TD, UK.*

**Summary** The controversial issue of enhanced pancreatic carcinogenesis following partial gastrectomy has been explored in male Wistar rats ($n = 40$) weighing 250–300 g. Animals were randomised to receive either 60% distal gastrectomy with Roux-en-Y reconstruction or gastrotomy and resuture (control). Immediately after operation each group was further divided into two subgroups, receiving i.p. injections of either saline or azaserine (30 mg $kg^{-1}$ $wk^{-1}$ for 3 weeks). At 15 months blood was obtained at 0, 5, 15 and 30 min after a fatty meal for cholecystokinin (CCK) assay; rats were then killed. Pancreatic wet weight was measured, and histological sections were examined for atypical acinar cell foci (AACF), the putative precursor lesion of carcinoma. There were no significant differences in body weight or pancreatic weight between controls and rats with gastrectomy. Only azaserine-treated rats had acidophilic AACF. Partial gastrectomy substantially increased the number of acidophilic AACF per pancreas (median 26.05 *vs* 2.09; $P < 0.005$), with a 9-fold increase in their volume ($P < 0.005$). Basal and postprandial plasma CCK concentrations were higher after gastrectomy than in controls ($P < 0.05$). Partial gastrectomy has an enhancing effect on azaserine-induced pancreatic carcinogenesis, probably by means of increased CCK release.

Since carcinoma of the pancreas is so difficult to cure and its aetiology remains obscure, it is important to investigate potential risk factors such as partial gastrectomy. Several reports have indicated an increased incidence of pancreatic cancer in patients undergoing gastric resection (Ross *et al.*, 1982; Mack *et al.*, 1986; Caygill *et al.*, 1987; Offerhaus *et al.*, 1987; Mills *et al.*, 1988; Tersmette *et al.*, 1990), but other work is contradictory (Maringhini *et al.*, 1987; Tomaszewska & Stachura, 1988; Vecchia *et al.*, 1990). The former popularity of partial gastrectomy for treating peptic ulcer disease, often in young patients, means that there are many patients alive today who could be at risk of cancer of the pancreas as well as cancer in the gastric stump (Schrumpf *et al.*, 1977).

In the alimentary canal, carcinogenesis can be enhanced by luminal factors acting directly on the mucosa to produce hyperplasia (Rainey *et al.*, 1984; Houghton *et al.*, 1987), but in the pancreas any such influence seems to be exerted indirectly through humoral and/or neural mechanisms. Cholecystokinin (CCK) promotes pancreatic carcinogenesis in the rat-azaserine model, in which the population of atypical acinar cell foci (AACF) of acidophilic type reflects the ultimate number of malignant tumours. Long-term administration of exogenous CCK increases the number of these preneoplastic AACF (Douglas *et al.*, 1989*a*), and the CCK antagonist CR-1409 blocks this effect (Douglas *et al.*, 1989*b*). The promoting effect of pancreatobiliary diversion on experimental pancreatic carcinogenesis may also be mediated through a sustained increase in circulating CCK (Stewart *et al.*, 1991, Watanapa *et al.*, 1991). Although partial gastrectomy does not increase fasting levels of CCK in man or the rat, the CCK response to ingested fat is markedly greater in both species (Hopman *et al.*, 1984; Inoue *et al.*, 1987; Malfertheiner *et al.*, 1987).

We have tested the hypothesis that partial gastrectomy enhances experimental pancreatic carcinogenesis, using quantitative estimation of AACF to show early malignant change and measuring CCK secretion to determine its intermediary role. We avoided any independent effect of duodenogastric reflux, which may itself enhance pancreatic carcinogenesis (unpublished data), by using Roux-en-Y reconstruction after partial gastrectomy rather than a Polya procedure.

Correspondence: R.C.N. Williamson, Department of Surgery, Hammersmith Hospital, RPMS, Du Cane Road, London W12 0NN, UK.

Received 11 September 1991; and in revised form 21 October 1991.

### Methods

### *Experimental design*

Male Wistar rats ($n = 40$) weighing 250–300 g were housed in groups of five in animal quarters with a 12 h day/night cycle. Standard pelleted rat food (Paterson and the Christopher Hill Group, Porton – diet PRD) and water were freely available. After 1 week of acclimatisation, animals were randomised to receive either 60% distal gastrectomy or gastrotomy and resuture (controls). Immediately after the operation, half the animals in each group were further randomised to receive saline or azaserine (see below). Food was reintroduced 12 h postoperatively. After overnight fasting, all rats were killed at 15 months after operation. Immediately before death rats were anaesthesised and a catheter was inserted into the inferior vena cava. Blood samples (2 ml) for CCK assay were obtained at 0, 5, 15 and 30 min after a fatty test meal comprising 3 ml soybean oil, 1 ml glucose solution (40%), 2 ml water and 0.4 g protein (Maxipro, Scientific Hospital Supplies, Liverpool, UK). The test meal was infused through a catheter placed just beyond the pylorus (in controls) or the gastric anastomosis (in rats with gastrectomy). The position of this catheter was chosen to avoid any effect of altered gastric emptying. Similar amounts of normal saline were infused intravenously to maintain the animals until the end of the test-meal study. After death, the pancreas was excised and trimmed free of adherent fat and lymph nodes. The wet weight of each gland was recorded before fixation in 10% buffered neutral formalin. Before immersion in the fixative solution, each pancreas was spread out on a piece of porous paper to ensure maximal transectional sectioning.

### *Operations* (Figure 1)

Partial gastrectomy was performed by removal of the distal 60% of the stomach. Gastrojejunostomy was carried out in a Roux-en-Y fashion with a 15 cm Roux loop of jejunum (roughly corresponding to the 45 cm loop usually created in man). Control rats received a gastrotomy, consisting of a 1 cm incision in the greater curvature of the stomach, which was immediately resutured. Operations were carried out

**Figure 1** Operations performed: 60% distal gastrectomy with Roux-en-Y anastomosis and gastrotomy with resuture (control).

under light ether anaesthesia, and a continuous 6 0 silk suture was used for anastomoses.

*Carcinogen*

Azaserine (Sigma Chemical Company, UK) was dissolved in 0.9% NaCl to give a 2.5% solution and was administered by weekly i.p. injection into each rat, starting immediately after operation and continuing for the next 2 weeks. The dosage regime was 30 mg $kg^{-1}$ $wk^{-1}$, giving a total dose of 90 mg $kg^{-1}$. Controls received 1.2 ml $kg^{-1}$ of 0.9% NaCl by weekly i.p. injection.

*CCK assay*

Plasma CCK peptides were extracted from venous blood samples with C18 'SepPak' cartridges (Waters, Harrow, UK) (Eysellein et al., 1987), and eluates were dried by centrifugal evaporation (Savant, Famingdale, NY, USA).

CCK was measured by a specific radioimmunoassay based on antiserum $A_2$, raised by immunising a rabbit with natural porcine CCK-33 (donated by Professors V. Mutt and S.R. Bloom). Antiserum $A_2$ (1:60 000) was incubated at 4°C for 3 days with standard CCK-8 or with plasma samples plus CCK-8 tracer-labelled with $^{125}$iodine (1,000 c.p.m., Amersham, UK) in 0.05 mol $l^{-1}$ sodium phosphate buffer (pH 7.4) containing 0.25% gelatin and 0.01 mol $l^{-1}$ EDTA. Free and bound tracer were separated by the addition of 6% (weight volume) charcoal (Norit PN5, BDH, Poole, UK) with 0.6% (weight volume) dextran. The concentrations of pure peptides that produced half-maximal inhibition of binding of tracer to $A_2$ were 2.0 pmol $l^{-1}$ for CCK-8, 2.4 pmol $l^{-1}$ for CCK-33, and 2.2 nmol $l^{-1}$ for gastrin 17. The coefficient of variation within assays was 8.2% and between assays 12.8%. The sensitivity of the assay (defined as minimal amount of CCK-8 that could be distinguished from zero with 95% confidence) was 0.2 pmol, and the recovery of CCK-8 and CCK-33 through the SepPak and assay procedure was 79%.

*Quantitative estimation of AACF*

The pancreas was spread out very thinly and then sectioned horizontally so that the whole gland could be examined. Histological sections (5 μM) of the whole pancreas were stained with haematoxylin and eosin, coded and scrutinised 'blind', i.e. the observers did not know what treatment each animal had received. The atypical acinar cell foci (AACF) were readily identified and classified as acidophilic or basophilic according to established criteria (Rao et al., 1982). The total area of exocrine pancreatic tissue was measured directly in a single histological section from each pancreas by means of a VIDS III video image analyser (Analytical Measuring Systems, Cambridge). The same instrument was used to count acidophilic and basophilic AACF and to measure their transectional area. Data were processed numerically by the Volugen computer package (InfoResearch Int., Bristol), using an algorithm based on that of Campbell et al. (Campbell et al., 1982) and modified by Pugh et al. (Pugh et al., 1983). Details of this analysis have been already described in our previous studies (Stewart et al., 1991).

*Statistical analysis*

Student's $t$-test for unpaired data was used for the group analysis of plasma CCK concentrations. The levels were expressed as means (SEM). Median values and ranges were quoted for body weight, pancreatic weight and quantitative estimation of AACF. Statistical analysis of these parameters were performed using Kruskal-Wallis one-way analysis of variance and the Mann-Whitney U-test.

### Results

*Mortality, body weight and pancreatic weight* (Table I)

There were five premature deaths from anastomotic leakage with granuloma formation and intestinal obstruction (two in gastrectomy-azaserine rats and one in each other group). Yields of healthy survivors were as follows: control-saline, nine; control-azaserine, nine; gastrectomy-saline, nine and gastrectomy-azaserine, eight.

There were no differences in body weight between the four groups. Although both absolute and relative pancreatic weights of rats with gastrectomy were greater than those of controls, the differences did not reach a significant level. Macroscopic examination of the pancreas at autopsy revealed numerous small white elevated nodules on the surface of the glands of azaserine-treated animals, particularly those with gastric resection.

*Plasma CCK* (Figure 2)

Partial gastrectomy increased basal circulating CCK concentrations by 46%. Following the test meal, the plasma CCK response at 5 min in rats with gastrectomy was greater than in controls (52% vs 41% increments over basal). At 15 and 30 min, plasma CCK levels remained 19% greater than those of controls, but these differences did not show statistical significance.

*Quantitative analysis of AACF*

No pancreatic carcinomas were found. Acidophilic AACF, the putative precancerous lesions, were only seen in azaserine-treated rats (Table II), whereas a few basophilic foci appeared in controls as well (Table III). Among azaserine-treated groups, the observed transectional data (foci per $cm^2$) revealed a marked increase in incidence of acidophilic lesions following partial gastrectomy compared with controls (2.29 vs 0.24). Quantitative stereological analysis of tissue sections confirmed the dramatic response of the pancreas to gastric resection with respect to acidophilic foci. Thus the number of lesions per cc. pancreas was substantially greater (15.40 vs 1.41), as was the total number of lesions per pancreas (26.05 vs 2.09). The median diameter of each lesion was increased by 69% and the volume by a factor of nine. Moreover, partial gastrectomy enhanced the percentage of the pancreatic volume occupied by acidophilic foci from 0.09% (control) to 3.38%. With regard to basophilic AACF, partial gastric resection increased the population of the lesions only with azaserine treatment, but the gastrectomised animals had fewer foci than the corresponding controls receiving saline.

### Discussion

Partial gastrectomy clearly promoted experimental pancreatic carcinogenesis, as shown by the very considerable increase in the number and size of acidophilic AACF. Acidophilic

**Table I** Body weight, absolute and relative pancreatic weight. Values are median (range)

| | *Control + saline* | *Control + azaserine* | *Gastrectomy + saline* | *Gastrectomy + azaserine* |
|---|---|---|---|---|
| Body weight (g) | 502 (355-529) | 520 (395-540) | 513 (438-527) | 512 (343-521) |
| Absolute pancreatic weight (mg) | 1350 (860-3000) | 1150 (920-1480) | 1550 (1000-3000) | 1880 (800-3550) |
| Relative pancreatic weight (mg/100 g body weight) | 292.16 (185.75-567.11) | 234.19 (216.98-278.20) | 331.05 (191.94-573.61) | 361.20 (157.80-1034.98) |

**Table II** Quantitative analysis of acidophilic atypical acinar cell foci (AACF). Values are medians (range)

| | *Control + saline* | *Control + azaserine* | *Gastrectomy + saline* | *Gastrectomy + azaserine* |
|---|---|---|---|---|
| No. of AACF $cm^{-2}$ | 0.00 | 0.24[a] (0.00-1.64) | 0.00 | 2.29[b,d] (0.39-6.82) |
| No. of AACF $cm^{-3}$ | 0.00 | 1.41[a] (0.00-14.43) | 0.00 | 15.40[b,c] (1.63-41.40) |
| No. of AACF pancreas | 0.00 | 2.09[a] (0.00-13.27) | 0.00 | 26.05[b,d] (4.31-123.37) |
| Mean focal diameter (μm) | 0.00 | 978.98[a] (0.00-1721.93) | 0.00 | 1650.23[b,d] (1094.54-2427.36) |
| Mean focal volume ($mm^3 \times 100$) | 0.00 | 35.52[a] (0.00-161.65) | 0.00 | 313.10[b,e] (147.95-551.96) |
| Volume as % of pancreas | 0.00 | 0.09[a] (0.00-0.98) | 0.00 | 3.38[b,e] (0.90-16.42) |

[a]$P < 0.05$. [b]$P < 0.001$. Significance *vs* control + saline or gastrectomy + saline groups. [c]$P < 0.05$. [d]$P < 0.005$. [e]$P < 0.001$. Significance *vs* control + azaserine group.

**Table III** Quantitative analysis of basophilic atypical acinar cell foci (AACF). Values are medians (range)

| | *Control + saline* | *Control + azaserine* | *Gastrectomy + saline* | *Gastrectomy + azaserine* |
|---|---|---|---|---|
| No. of AACF $cm^{-2}$ | 0.00 (0.00-5.06) | 0.00 (0.00-2.15) | 0.00[b] | 0.12[a] (0.00-2.81) |
| No. of AACF $cm^{-3}$ | 0.00 (0.00-116.51) | 0.00 (0.00-70.16) | 0.00[b] | 2.51[a] (0.00-64.48) |
| No. of AACF pancreas | 0.00 (0.00-157.29) | 0.0 (0.00-103.84) | 0.00[b] | 2.60[a] (0.00-103.17) |
| Mean focal diameter (μm) | 0.00 (0.00-678.08) | 0.00 (0.00-512.92) | 0.00[b] | 135.00[a] (0.00-774.83) |
| Mean focal volume ($mm^3 \times 100$) | 0.00 (0.00-12.06) | 0.00 (0.00-6.35) | 0.00[b] | 0.28[a] (0.00-14.61) |
| Volume as % of pancreas | 0.00 (0.00-0.92) | 0.00 (0.00-0.28) | 0.00[b] | 0.01[a] (0.00-0.29) |

[a]$P < 0.05$. Significance *vs* gastrectomy + saline group. [b]$P < 0.05$. Significance *vs* control + saline group.

AACF are well established as the precursors of cancer in this model (Rao *et al.*, 1982; Roebuck *et al.*, 1984), and they were only found in rats receiving azaserine. Acidophilic AACF show considerable growth potential with a mitotic index (2.75) which greatly exceeds that of basophilic foci (0.125) or normal pancreas (zero) (Scarpelli *et al.*, 1984). Their increased number after gastrectomy mirrored our subjective

**Figure 2** Plasma cholecystokinin levels (pmol $l^{-1}$) following a fatty test meal (mean values shown with standard errors of the mean).

assessment that there were many more macroscopic nodules on the surface of the pancreas in these rats. We encountered fewer AACF in all groups compared with Longnecker's reports (Longnecker *et al.*, 1977; Roebuck *et al.*, 1985) and our own previous experience (Stewart *et al.*, 1991), probably because of the relatively large size of rat chosen to facilitate the gastric operation. Likewise, no actual carcinomas were found in the pancreas, although Longnecker and Curphey reported a few of these lesions at 1 year when much younger rats were given azaserine (Longnecker & Curphey, 1975). Although quantitative analysis showed that the population of basophilic foci was also increased in both number and size after partial gastrectomy, the relevance of this finding is doubtful since most modulators of the postinitiation phase of pancreatic carcinogenesis have little effect on basophilic foci (Roebuck *et al.*, 1982; Roebuck *et al.*, 1985).

Partial gastrectomy alters circulating levels of several gut peptides, notably gastrin, pancreatic polypeptide and CCK (Inoue *et al.*, 1987; Malfertheiner *et al.*, 1987; Rieu *et al.*, 1990). Exogenous gastrin stimulates pancreatic growth (Johnson, 1976), whereas pancreatic polypeptide inhibits pancreatic secretion (Taylor *et al.*, 1979); the effect of reducing their circulating levels has not been established in pancreatic carcinogenesis. The increase in basal CCK concentrations and the increased CCK response to a fatty test meal strongly implicate this peptide as an intermediary in the promoting effect of partial gastrectomy. The enhanced postprandial

CCK response is in line with other reports both in man and the rat (Hopman et al., 1984; Inoue et al., 1987; Malfertheiner et al., 1987), but unlike other authors we also found a higher basal level. Previous studies were undertaken either 2 weeks after partial gastrectomy in rats or 1 month after partial gastrectomy in man; our data suggest that hypercholecystokininaemia persists for up to 15 months and may even increase with time.

Since direct infusion of the fatty meal into the small bowel circumvented any variability in gastric emptying, the increased CCK release after partial gastrectomy may be due to an increased responsiveness of CCK-secreting cells. Diversion of pancreatobiliary secretions from the jejunal limb of a Roux-en-Y anastomosis can cause mucosal hyperplasia (Miazza et al., 1982), and this hyperplastic response might well involve the enteroendocrine cells and lead to increased cholecystokinin production. Lower concentrations of intraluminal protease have been shown in patients with subtotal gastric resection in the early phase after a fatty meal (MacGregor et al., 1977). Similar protease reduction in the rat might also contribute to an increased CCK response, since low levels of intraluminal trypsin are known to stimulate CCK release (Louie et al., 1986; Calam et al., 1987). Although CCK stimulates pancreatic growth, the fact that there is a loss of the normal pancreatic response to several tropic hormones (including CCK) with advancing age (Greenberg et al., 1986; Poston et al., 1991) might explain the non-significant increase in pancreatic weight 15 months after partial gastrectomy.

Data from an experimental rat model can only be of tentative relevance to man. Although our rats received a carcinogen, patients with previous partial gastrectomy have increased levels of nitrites and n-nitroso compounds in gastric juice, and these substances can act as pancreatic carcinogens (Schlag et al., 1980). Nitrosamines could be absorbed and subsequently secreted into the pancreatic juice or might reflux from the duodenum into the pancreatic duct, thereby inducing pancreatic cancer. The combination of increased postprandial CCK release and greater exposure to pancreatic carcinogens might explain the increase in pancreatic cancer risk after gastrectomy. Our unpublished data showing only a few acidophilic AACF 6 months after a similar partial gastrectomy in rats underline the importance of a long-term experiment for a clear-cut effect to emerge. They could also explain why an increased risk of pancreatic cancer in man only appears to reach statistical significance 20 years after gastric resection (Caygill et al., 1987; Tersmette et al., 1990).

We thank the Royal Postgraduate Medical School and the Hammersmith and Queen Charlotte's Special Health Authority for supporting this research.

### References

CALAM, J., BOJARSKI, J.C. & SPRINGER, C.J. (1987). Raw soya-bean flour increases cholecystokinin release in man. *Br. J. Nutr.*, **58**, 175.

CAMPBELL, H.A., PITOT, H.C., POTTER, B.R. & LAISHES, B.A. (1982). Applications of quantitative stereology to the evaluation of enzyme altered foci in rat liver. *Cancer Res.*, **42**, 465.

CAYGILL, C.P.J., HILL, M.J., HALL, C.N., KIRKHAM, J.S. & NORTHFIELD, T.C. (1987). Increased risk of cancer at multiple sites after gastric surgery for peptic ulcer. *Gut*, **28**, 924.

DOUGLAS, B.R., WOUTERSEN, R.A., JANSEN, J.B.M.J., DE JONG, A.J.L., ROVATI, L.C. & LAMERS, C.B.H.W. (1989*a*). Influence of cholecystokinin antagonist on the effects of cholecystokinin and bombesin on azaserine-induced lesions in rat pancreas. *Gastroenterology*, **96**, 462.

DOUGLAS, B.R., WOUTERSEN, R.A., JANSEN, J.B.M.J., DE JONG, A.J.L., ROVATI, L.C. & LAMERS, C.B.H.W. (1989*b*). Modulation by CR-1409 (Lorglumide), a cholecystokinin receptor antagonist, of trypsin inhibitor-enhanced growth of azaserine-induced putative preneoplastic lesions in rat pancreas. *Cancer Res.*, **49**, 2438.

EYSELLEIN, V.E., EBERLEIN, G.E., HESSE, W.H., SUGER, M.V., GOEBELL, H. & REEVE, J.R. (1987). Cholecystokinin-58 is the major circulating form of cholecystokinin in canine blood. *J. Biol. Chem.*, **262**, 214.

GREENBERG, R.E., DOMINGUEZ, A., WASHINGTON, A. & HOLT, P.R. (1986). Impaired response of aging rat pancreas to cerulein-secretin stimulation. *Gastroenterology*, **90**, 1438.

HOPMAN, W.P.M., JANSEN, J.B.M.J. & LAMERS, C.B.H.W. (1984). Plasma cholecystokinin response to oral fat in patients with Billroth I and Billroth II gastrectomy. *Ann. Surg.*, **199**, 276.

HOUGHTON, P.W.J., MORTENSEN, N.J.MCC. & WILLIAMSON, R.C.N. (1987). Effect of duodenogastric reflux on gastric mucosal proliferation after gastric surgery. *Br. J. Surg.*, **74**, 288.

INOUE, K., FUCHIGAMI, A., HOSOTANI, R. & 9 others (1987). Release of cholecystokinin and gallbladder contraction before and after gastrectomy. *Ann. Surg.*, **205**, 27.

JOHNSON, L.R. (1976). The trophic action of gastrointestinal hormones. *Gastroenterology*, **70**, 278.

LONGNECKER, D.S. & CURPHEY, T.J. (1975). Adenocarcinoma of the pancreas in azaserine-treated rats. *Cancer Res.*, **35**, 2249.

LONGNECKER, D.S., FRENCH, J., HYDE, E., LILJA, H.S. & YAGER, J.D. Jr (1977). Effect of age on nodule induction by azaserine and DNA synthesis in rat pancreas. *JNCI*, **58**, 1769.

LOUIE, D.S., MAY, D., MILLER, P. & OWYANG, C. (1986). Cholecystokinin mediates feedback regulation of pancreatic enzyme secretion in rats. *Am. J. Physiol.*, **250**, G252.

MACGREGOR, I., PARENT, J. & MEYER, J.H. (1977). Gastric emptying of liquid meals and pancreatic biliary secretion after subtotal gastrectomy or truncal vagotomy and pyloroplasty in man. *Gastroenterology*, **72**, 195.

MACK, T.M., YU, M.C., HANISCH, R. & HENDERSON, B.E. (1986). Pancreas cancer and smoking, beverage consumption, and past medical history. *JNCI*, **76**, 49.

MALFERTHEINER, P., BUCKLER, M., GLASBRENNER, B., SCHAFMAYER, A. & DITSCHUNEIT, H. (1987). Adaptive changes of the exocrine pancreas and plasma cholecystokinin release following subtotal gastric resection in rats. *Digestion*, **38**, 142.

MARINGHINI, A., THIRUVENGADAM, R., MELTON, L.J. III, HENCH, V.S., ZINSMEISTER, A.R. & DIMAGNO, E.P. (1987). Pancreatic cancer risk following gastric surgery. *Cancer*, **60**, 245.

MIAZZA, B.M., VAN HUNG, L., VAJA, S. & DOWLING, R.H. (1982). Effects of pancreatobiliary diversion (PBD) on jejunal and ileal structure and function in the rat. In Robinson, J.W.L., Dowling, R.H. & Riecken, E.-O. (eds) *Mechanisms of Intestine Adaptation*. Lancaster: MTP Press, pp. 467.

MILLS, P.K., BEESON, W.L., ABBEY, D.E., FRASER, G.E. & PHILLIPS, R.L. (1988). Dietary habits and past medical history as related to fatal pancreas cancer risk among adventists. *Cancer*, **61**, 2578.

OFFERHAUS, G.J.A., GIARDIELLO, F.M., MOORE, G.W. & TERSMETTE, A.C. (1987). Partial gastrectomy: a risk factor for carcinoma of the pancreas? *Hum. Pathol.*, **18**, 285.

POSTON, G.J., SAYDJARI, R., LAWRENCE, J.P., CHUNG, D., TOWNSEND, C.M. Jr & THOMPSON, J.C. (1991). Aging and trophic effects of cholecystokinin, bombesin and pentagastrin on the rat pancreas. *Pancreas*, **6**, 407.

PUGH, T.D., KING, J.H., KOEN, H. & 5 others (1983). Reliable stereological method for estimating the number of microscopic hepatocellular foci from their transections. *Cancer Res.*, **43**, 1261.

RAINEY, J.B., DAVIES, P.W. & WILLIAMSON, R.C.N. (1984). Relative effects of ileal resection and bypass on intestinal adaptation and carcinogenesis. *Br. J. Surg.*, **71**, 197.

RAO, M.S., UPTON, M.P., SUBARAO, V. & SCARPELLI, D.G. (1982). Two populations of cells with differing proliferative capacities in atypical acinar foci induced by 4-hydroxyaminoquinolone-1-oxide in the rat pancreas. *Lab. Invest.*, **46**, 527.

RIEU, P.N.M.A., JANSEN, J.B.M.J., HOPMAN, W.P.M., JOOSTEN, H.J.M. & LAMERS, C.B.H.W. (1990). Effect of partial gastrectomy with Billroth II or Roux-en-Y anastomosis on postprandial and cholecystokinin-stimulated gallbladder contraction and secretion of cholecystokinin and pancreatic polypeptide. *Dig. Dis. Sci.*, **35**, 1066.

ROEBUCK, B.D., BAUMGARTNER, K.J. & THRON, C.D. (1984). Characterisation of two populations of pancreatic atypical acinar cell foci induced by azaserine in the rat. *Lab. Invest.*, **50**, 141.

ROEBUCK, B.D., LONGNECKER, D.S., BAUMGARTNER, K.J. & THRON, D.C. (1985). Carcinogen-induced lesions in the rat pancreas: effects of varying levels of essential fatty acid. *Cancer Res.*, **45**, 5252.

ROSS, A.H.M., SMITH, M.A., ANDERSON, J.R. & SMALL, W.P. (1982). Late mortality after surgery for peptic ulcer. *N. Engl. J. Med.*, **307**, 519.

SCARPELLI, D.G., RAO, M.S. & REDDY, J.K. (1984). Studies of pancreatic carcinogenesis in different animal models. *Environ. Health Perspect.*, **56**, 219.

SCHLAG, P., ULRICH, H., MERKLE, P., BOCKLER, R., PETER, M. & HERFARTH, C. (1980). Are nitrite and n-nitroso compounds in gastric juice risk factors for carcinoma in the operated stomach? *Lancet*, **i**, 727.

SCHRUMPF, E., STADASS, J., MYREN, J., SERCK-HANSSEN, A., AUNE, S. & OSNES, M. (1977). Mucosal changes in the gastric stump 20 to 25 years after partial gastrectomy. *Lancet*, **ii**, 467.

STEWART, I.D., FLAKS, B., WATANAPA, P., DAVIES, P.W. & WILLIAMSON, R.C.N. (1991). Pancreatobiliary diversion enhances experimental pancreatic carcinogenesis. *Br. J. Cancer*, **63**, 63.

TAYLOR, I.L., SOLOMON, T.E., WALSH, J.H. & GROSSMAN, M.I. (1979). Pancreatic polypeptide, metabolism and effect on pancreatic secretion in dogs. *Gastroenterology*, **76**, 524.

TERSMETTE, A.C., OFFERHAUS, J.A., GIARDIELLO, F.M., TERSMETTE, K.W.F., VANDERBROUCKE, J.P. & TYTGAT, G.N.J. (1990). Occurrence of non-gastric cancer in the digestive tract after remote partial gastrectomy: analysis of an Amsterdam cohort. *Int. J. Cancer*, **46**, 792.

TOMASZEWSKA, R. & STACHURA, J. (1988). Gastrectomy – a risk factor for pancreatic carcinoma? *Hum. Pathol.*, **19**, 491.

VECCHIA, C.L., NEGRI, E., D'AVANZO, B. & 5 others (1990). Medical, history, diet and pancreatic cancer. *Oncology*, **47**, 463.

WATANAPA, P., EFA, E.F., BEARDSHALL, K. & 4 others (1991). Inhibitory effect of a cholecystokinin antagonist on the proliferative response of the pancreas to pancreatobiliary diversion. *Gut*, **32**, 1049.